# Bureau of Public Health

**Published:** 1910-01

**Authors:** 


A Monthly Review of Medicine and Surgery.

*EDITOR*

WILLIAM WARREN POTTER, M. D.

All communications, whether of a literary or business nature, books for review and xchanges, should be addressed to the editor, 238 Delaware Avenue, Buffalo, N.Y

Vol. Lxv. JANUARY, 1910. No. 6

### Bureau of Public Health

FOR some years past the establishment of a department of health, to be presided over by a cabinet officer, has been advocated by a large number of prominent medical men. It is the opinion of many sanitarians and others who have given the subject much thought, that our present methods of supervision of the public health are inadequate, and that enlarged powers should be conferred upon a single administrative officer who should be a member of the President's official family.

There is a little question of the propriety of improving present conditions, which fail to meet the requirements of our rapid growth, increasing importance and great numerical strength. It may not be easy to determine just what the remedy should be, but it is well to discuss the question in journals and societies with a view to reach a solution of a problem so important as the conservation of the health of the people of the United States.

The President, in his recent annual message to congress, presents the subject for legislative consideration with much force and in considerable detail. President Taft, however, does not recommend the creation of a department of public health to be presided over by a cabinet minister, but suggests a merger of the offices and bureaus that have to do with the public health and subjects akin thereto, into one general bureau to be known as the "Bureau of Public Health." We extract from the message what the President says on the subject.

BUREAU OF HEALTH.

> For a very considerable period a movement has been gathering strength, especially among the members of the medical profession, in favor of a concentration of the instruments of the national government which have to do with the promotion of public health. In the nature of things, the medical department of the army and the medical department of the navy must be kept separate. But there

> seems to be no reason why all the other bureaus and offices in the general government which have to do with the public health or subjects akin thereto should not be united in a bureau, to be called the "Bureau of Public Health." This would necessitate the transfer of the Marine Hospital Service to such a bureau. I am aware that there is a wide field in respect to the public health committed to the states in which the federal government cannot exercise jurisdiction, but we have seen in the Agricultural Department the expansion into widest usefulness of a department giving attention to agriculture when that subject is plainly one over which the states properly exercise direct jurisdiction. The opportunities offered for useful research and the spread of useful information in regard to the cultivation of the soil and the breeding of stock and the solution of many of the intricate problems in progressive agriculture have demonstrated the wisdom of establishing that department. Similar reasons, of equal force, can be given for the establishment of a bureau of health that shall not only exercise the police jurisdiction of the federal government respecting quarantine, but which shall also afford an opportunity for investigation and research by competent experts into questions of health affecting the whole country, or important sections thereof, questions which in the absence of federal governmental work, are not likely to be promptly solved.

We are not quite sure that the establishment of such a bureau will remedy the defects inherent in present methods, though we are pretty certain it will not satisfy the advocates of the department plan.

Notwithstanding these objections it may be wise for congress to provide for a bureau at first and thus test the measure. Should it prove inadequate, it will be easy, comparatively speaking, to raise the bureau to a department.

